# Patterns of physical activity and their relationship with depression among community-dwelling older adults in Shanghai, China: a latent class approach

**DOI:** 10.1186/s12877-021-02537-8

**Published:** 2021-10-21

**Authors:** Yan Liang, Xinghui Li, Tingting Yang, Mengying Li, Ye Ruan, Yinghua Yang, Yanyan Huang, Yihua Jiang, Ying Wang

**Affiliations:** 1 School of Nursing, Fudan University, Shanghai, 200032 China; 2 Fudan University School of Public Health, Shanghai, 200032 China; 3 Shanghai Center for Disease Control and Prevention, Shanghai, 200336 China; 4 Shanghai Center for Clinical Laboratory, Shanghai, 200126 China; 5 Department of Geriatrics, Huashan Hospital Fudan University, Shanghai, 200040 China; 6 TianQiao and Chrissy Chen Institute Clinic Translational Research Center, Shanghai, 200040 China; 7 Shanghai Medicine-Mental Health Center of Minhang District, 130 DongAn Road, Shanghai, 200032 China; 8 Minhang Branch, School of Public Health, Fudan University, 130 DongAn Road, Shanghai, 200032 China; 9 Key Laboratory of Health Technology Assessment, National Health and Family Planning Commission of the People’s Republic of China, Fudan University, 130 DongAn Road, Shanghai, 200032 China

**Keywords:** Physical activity, Patterns, Depression, Latent class analysis, LCA, Older adults

## Abstract

**Background:**

Few studies have explored patterns of physical activity (PA) and examined their relationship with depression among community-dwelling older adults. We aimed to identify the patterns of PA through a person-centered analytical approach and examine the association between quantity and patterns of PA, and depression among community-dwelling older adults.

**Methods:**

We conducted a cross-sectional survey study in the Minhang district, Shanghai, China, in August 2019, and used a self-administered questionnaire to collect data through home visits. The total sample included 2525 older adults. This study used the Physical Activity Scale for the Elderly (PASE) to assess the quantity of PA in older adults. Depression was evaluated with the Geriatric Depression Scale (GDS). Latent class analysis (LCA) was used to identify subpopulations by shared item response patterns. Logistic regressions were performed to estimate the relationship between PASE score, patterns of PA, and depression. An exploratory analysis of joint levels and patterns of PA effects on depression was based on sample subgroups with combinations of levels and patterns of PA. Logistic regression was used to calculate the odds ratio for combined subgroups.

**Results:**

Four latent classes were identified: “domestic types,” “athletic types,” “gardening/caring types,” and “walkers.” PASE scores and patterns of PA both were associated with depression. Older adults who were the most active (PASE quartile: 75–100%) and the athletic types had the strongest significant association with depression (*OR =* 0.19, 95% CI: 0.06–0.65), followed by those who were the most active (PASE quartile: 75–100%) and the walkers (*OR* = 0.28, 95% CI: 0.14–0.57) when compared with older adults with the least activity (PASE quartile: 0–25%) and domestic types.

**Conclusion:**

This study suggests both the quantity and patterns of physical activity are associated with depressive symptoms among community-dwelling older adults. Population-level intervention should encourage community-dwelling older adults to increase their quantity of PA to reduce the risk of depression. Athletics and walkers are recommended. To develop individual-level tailored interventions, more attention should be paid to older adults who are highly engaged in gardening/caring for others.

**Supplementary Information:**

The online version contains supplementary material available at 10.1186/s12877-021-02537-8.

## Background

Depression is one of the most prevalent mental disorders in later life that has a high risk of disability worldwide [1–3]. Depression and depressive symptoms are attracting considerable interests due to their related consequences, including increased chronic disease [4], suicide and non-suicide mortality [5], and high disease burden [6].

Physical activity (PA) may be beneficial in reducing the risk of depression, but studies have reported mixed results [7–14]. The intensity, frequency, duration, volume, and types of physical activity may contribute to the inconsistency [15]. Previous studies have focused on the quantity or levels of PA and their relationship with depression in older people [16, 17]. Some studies showed that PA with higher frequency or moderate-to-vigorous PA were associated with lower odds of depression [18, 19]; some suggested that light physical activity or lower frequency were protective [20, 21]. Recent research has shown that different patterns of PA may have an influence on depression, as some patterns of PA may be protective against depression for older adults while others may not [22, 23]. For example, daily purposeful exercise (walking, tai chi, aerobic/strength training) showed a stronger relationship with depression than domestic or transportation-related PA [8, 9, 22, 23]. Patterns of PA can provide more comprehensive information than the quantity or levels of PA. Research showed that high levels of PA across multiple domains, or athletic pattern were at lower risk for depression [22]. A key unanswered question is whether certain patterns of PA are particularly associated with decreased risk of depression at certain levels of PA. Therefore, knowledge about the joint effects of patterns of PA and levels of PA on depression risk would be helpful.

Patterns of PA include work-related (e.g. work for pay or as a volunteer), domestic-related (e.g. housework), and leisure-time activity (e.g. walking outside home) [24]. Previous studies in Western countries showed that leisure-time PA had a stronger inverse relationship with depression than other patterns of PA [22–24]. It is important to understand the contribution of specific patterns of PA on depression so as to develop appropriate public health recommendations. The association between patterns of PA and depression depends largely on the specific cultural contexts [25]. East Asians tend to take fewer physical activities and at lower intensity than those in Western countries [26], and may present distinctive patterns of PA.

It is challenging to compare the benefits of different patterns of PA as they are highly correlated; for example, older adults who do domestic work may be more likely to garden [22]. Latent class analysis (LCA) provides a person-centered approach for identification of patterns of PA and has been used in several studies [27–29]. To our knowledge, there has been no study exploring patterns of PA using LCA and examining the relationship between patterns of PA and depression among Chinese community-dwelling older adults. Our study may provide implications for developing community interventions in Eastern culture, both to support some patterns of PA and to target specific latent classes of community-dwelling older adults.

The purposes of this study were to: (1) identify the patterns of PA among Chinese community-dwelling older adults; (2) examine the independent contribution that quantity and patterns of PA have on depression among Chinese community-dwelling older adults; and (3) explore the joint effects of levels and patterns of PA on depression among Chinese community-dwelling older adults.

## Methods

### Participants

**Fig. 1:**
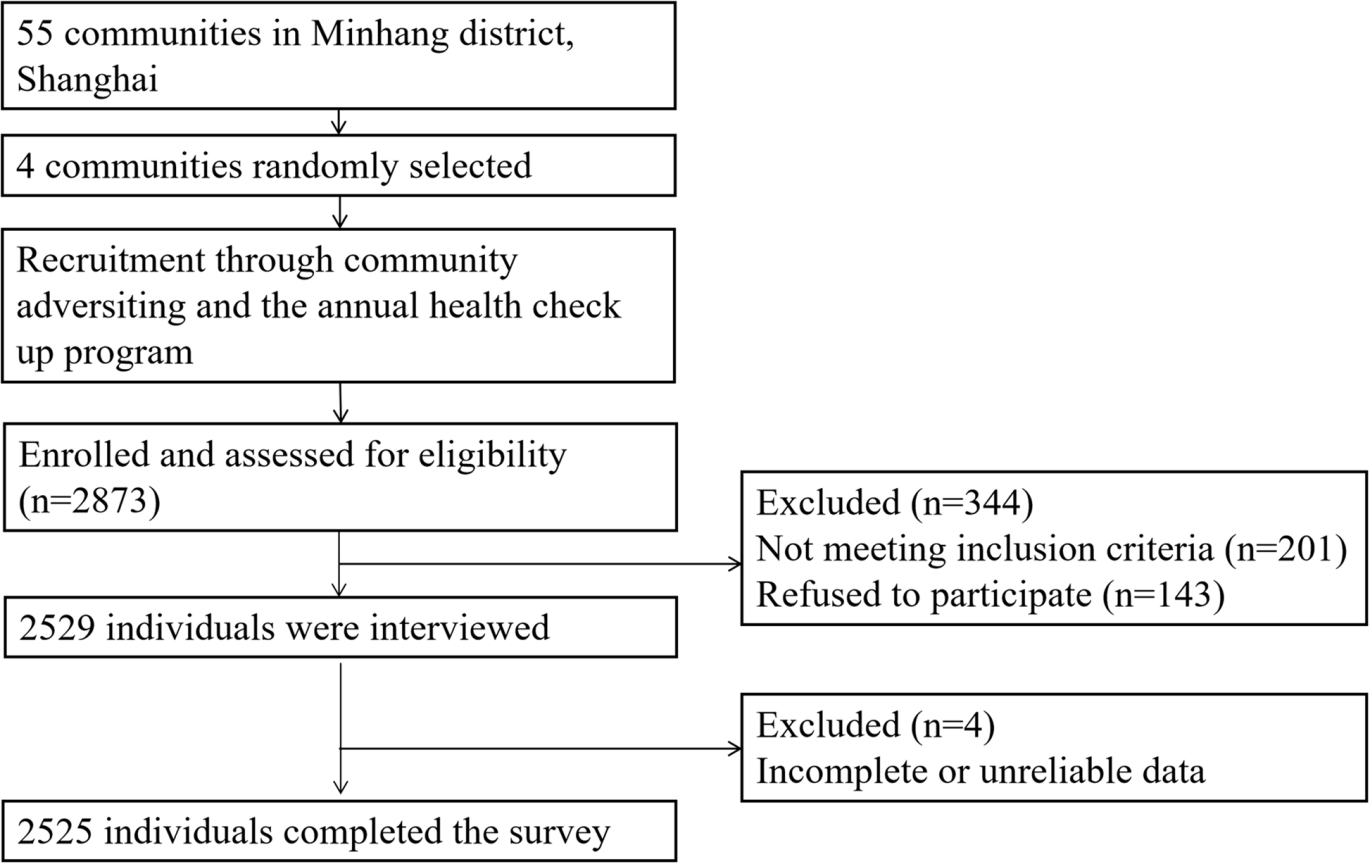
Flow diagram of the selection of eligible participants

A cross-sectional survey study was conducted in the Minhang district, Shanghai, China, in August 2019. We used a self-administered questionnaire to collect data through home visits. The survey was performed by trained investigators and data were collected through face-to-face interview. The sample was randomly selected from the census database of older adults in the Minhang district, Shanghai. A stratified cluster random sampling design was used and four communities were selected based on geographical area, sex, and age. Participants were recruited with the annual health check up programs. Inclusion criteria were: 1) age ≥ 60; 2) being community-dwelling; and 3) being able to communicate and willing to consent and participate. Exclusion criteria were as follows: 1) inability to understand and follow the assessment protocol of the study; and 2) having major neurocognitive disorders, such as dementia (confirmed by community doctors based on the health information). The total sample included 2525 older adults (see Fig. 1). This research protocol was approved by the Ethical Review Board of Fudan University (reference number: IRB#TYSQ 2019-2-03), and informed written consent was obtained before data collection.

### Measures

#### Physical activity

This study used the Physical Activity Scale for the Elderly (PASE) to assess the quantity of PA in older adults. All participants were asked to recall past-week leisure time activity, household activity, and paid or volunteer work. Items included engagement in walking, sports and recreation, muscle-strengthening and endurance exercises, housework, home repair, gardening, yard work, caring for others, and paid or volunteer work [30]. For leisure time activity, individuals responded “never,” “rarely,” “sometimes,” or “often.” The average time spent on each type of activity per day was also recorded. For household activity and paid or volunteer work, participants responded “yes” or “no.” Information on the hours and types of work involved was also gathered. Each PASE item had a weight value determined by the original authors of the measurement tool [31]. The overall PASE score was calculated by multiplying the amount of time spent and participation by the PASE weight value, and then summing for a total score. The PASE scores ranged from 0 to 500 or more. The higher the scores, the higher the PA levels. PASE scores have been validated against several objective activity measures, including accelerometers and doubly labeled water [32, 33]. There is evidence supporting the validity and reliability of the Chinese version of the PASE [34].

Indicators of activity types were developed from PASE items and two modifications were made based on the study of Mooney et al. [35]. We excluded an item assessing physical activity from employment as this population were mostly retired. First, to retain information regarding activity duration in categorical variables used by LCA, we recoded PASE items indicating the duration of activity (walking, sports and recreation, and muscle-strengthening exercises) into dichotomous variables indicating ever having engaged (> 0 min/day on any day) in the activity and often engaging (> 30 min/day on average). Second, because many subjects reported participation in a major sport/recreation activity, exploratory LCAs that using the original separately coded items resulted in patterns of PA that were strongly influenced by intensity of a single sport/recreation activity rather than an overall pattern of activity. We combined all sport/recreation categories for analysis, so as to increase homogeneity of the classes and optimize interpretability of the patterns. Sports or recreational activities such as dancing, hiking, and jogging were classified as sports, and muscular strength or endurance exercises (such as sit-ups and weight lifting) were classified as exercise. Finally, a total of 12 items were used in the LCA model including the following items: ever does sports, ever exercises, ever walks, often-sports, often-exercises, often-walks, light housework, heavy housework, home repairs, yard care, outdoor gardening, and caring for others.

### Depressive symptoms

The 30-item Geriatric Depression Scale (GDS), used worldwide, was utilized to assess the depressive symptoms of the participants [36]. Participants were asked to respond “yes” or “no” to each item. Summary scores ranged from 0 to 30. A well-validated cutoff point ≥11 was used to define depression [37]. The validity and reliability of the GDS-30 has been tested in China and also used in Chinese older adults [38].

### Sociodemographic factors and physical health status

Participants’ sociodemographic characteristics were measured as follows (see the additional file 1): (1) age (categorized as 60–74 = 1, 75–84 = 2, ≥ 85 = 3); (2) sex (male = 0, female = 1); (3) marital status (married = 1, others = 0); (4) educational background (illiteracy = 1, primary school = 2, middle school = 3, high school = 4, college and more = 5); (5) income (≤ 2000 = 1, 2001–5000 = 2, ≥ 5001 = 3); and (6) living arrangements (living alone = 1, live with spouse only = 2, live with spouse and children = 3, live with others = 4).

Physical health status was assessed by two variables (see the additional file 1): (1) having any chronic disease (Yes = 1, No = 0), and (2) self-rated health. To measure self-rated health, each participant was asked, “How would you describe your current health status?” The responses ranged from 1 to 5 indicating excellent to poor health. We reverse coded self-rated health to make higher values indicate better health so that the results were easier to interpret.

### Statistical analysis

Descriptive statistics were used to summarize sample characteristics. LCA was used to identify subpopulations (latent classes) by shared item response patterns. LCA was conducted using the maximum likelihood estimation with robust standard errors. A series of LCA models with a successive number of classes were specified and selection of the optimal model that combines goodness of fit and parsimony was based on conceptual considerations and various statistical fit indices [39]. Logistic regressions were performed to estimate the relationship between PASE score, patterns of PA, and depression. In multivariate analyses, levels and patterns of PA were included simultaneously in the same model to estimate their independent contributions to depression risk. All models were adjusted for age, sex, education, income, living arrangements, self-rated health, and chronic disease. An exploratory analysis of joint levels and patterns of PA effects on depression was based on sample subgroups with combinations of levels and patterns of PA. Logistic regression was used to calculate the odds ratio for combined subgroups. LCA models were conducted in Mplus version 8.0 and all subsequent analyses were performed using Stata SE version 15.1(StataCorp., College Station, TX, USA).

## Results

### Participant characteristics

Table 1 presents the participants’ characteristics and group differences among four patterns of PA. The majority of the sample was age 60–74 years (*N* = 1829, 72.4%), 55.8% (*N* = 1410) were female, and 83.6% (*N* = 2111) were married. The average PASE score was 119.34 (SD = 41.93). A minority (13.4%) reported depression. Significant differences were found among groups of four patterns of PA in all characteristics expect income.

**Table 1:** Participants characteristics and group differences among four patterns of PA (*N* = 2525)

Characteristics	Whole sample (*n* = 2525) n (%) or mean ± *SD*	Domestic types (*n* = 751)	Athletic types (*n* = 295)	Gardening/Caring types (*n* = 528)	Walkers (*n* = 951)	*P* Value
		n (%) or mean ± *SD*	n (%) or mean ± *SD*	n (%) or mean ± *SD*	n (%) or mean ± *SD*	
**Age group (years)**						< 0.001
*60–74*	1829 (72.4)	575 (76.6)	235 (79.7)	227 (43.0)	792 (83.3)	
*75–84*	503 (19.9)	147 (19.6)	55 (18.6)	169 (32.0)	132 (13.9)	
*≥ 85*	193 (7.7)	29 (3.9)	5 (1.7)	132 (25.0)	27 (2.8)	
**Gender**						< 0.001
*Male*	1115 (44.2)	296 (39.4)	122 (41.4)	270 (51.1)	427 (44.9)	
*Female*	1410 (55.8)	455 (60.6)	173 (58.6)	258 (48.9)	524 (55.1)	
**Education**						< 0.001
*Illiteracy*	194 (7.7)	44 (5.9)	11 (3.7)	81 (15.3)	58 (6.1)	
*Primary school*	463 (18.3)	142 (18.9)	34 (11.5)	119 (22.5)	168 (17.7)	
*Middle school*	901 (35.7)	282 (37.5)	112 (38.0)	153 (29.0)	354 (37.2)	
*High school*	658 (26.1)	191 (25.4)	89 (30.2)	115 (21.8)	263 (27.7)	
*College and more*	309 (12.2)	92 (12.3)	49 (16.6)	60 (11.4)	108 (11.4)	
**Marital status**						< 0.001
*Married*	2111 (83.6)	623 (83.0)	260 (88.1)	394 (74.6)	834 (87.7)	
*Others*	414 (16.4)	128 (17.0)	35 (11.9)	134 (25.4)	117 (12.3)	
**Income (Yuan)**						0.014
*≤ 2000*	327 (12.9)	109 (14.5)	20 (6.8)	71 (13.5)	127 (13.3)	
*2001–5000*	1757 (69.6)	503 (67.0)	231 (78.3)	363 (68.8)	660 (69.4)	
*≥ 5001*	441 (17.5)	139 (18.5)	44 (14.9)	94 (17.8)	164 (17.3)	
**Living arrangements**						< 0.001
*Living alone*	199 (7.9)	75 (10.0)	18 (6.1)	47 (8.9)	59 (6.2)	
*Live with spouse only*	1381 (54.7)	385 (51.3)	180 (61.0)	269 (51.0)	547 (57.5)	
*Live with spouse and children*	459 (18.2)	140 (18.6)	53 (18.0)	83 (15.7)	183 (19.2)	
*Live with others*	486 (19.2)	151 (20.1)	44 (14.9)	129 (24.4)	162 (17.0)	
**Self-rated health**	2.90 ± 0.99	2.95 ± 0.98	3.13 ± 0.96	2.43 ± 0.96	3.05 ± 0.93	< 0.001
**Chronic disease**						< 0.001
*Yes*	1652 (65.4)	493 (65.7)	169 (57.3)	412 (78.0)	578 (60.8)	
*No*	873 (34.6)	258 (34.3)	126 (42.7)	116 (22.0)	373 (39.2)	
**PASE score**	119.3 ± 41.9	93.6 ± 31.9	144.5 ± 60.2	132.0 ± 27.4	124.8 ± 38.6	< 0.001
**PASE quartile**						< 0.001
*0–25%*	642 (25.4)	433 (57.7)	28 (9.5)	18 (3.4)	163 (17.1)	
*25–50%*	627 (24.8)	154 (20.5)	58 (19.7)	98 (18.6)	317 (33.3)	
*50–75%*	631 (25.0)	109 (14.5)	86 (29.1)	232 (43.9)	204 (21.5)	
*75–100%*	625 (24.8)	55 (7.3)	123 (41.7)	180 (34.1)	267 (28.1)	
**GDS score**						< 0.001
*< 11*	2186 (86.6)	651 (86.7)	285 (96.6)	386 (73.1)	864 (90.8)	
*≥ 11*	339 (13.4)	100 (13.3)	10 (3.4)	142 (26.9)	87 (9.2)	

Notes: One-way ANOVA was used for continuous variables, and chi-square test was used for categorical variables to explore differences among four patterns of PA: domestic types, athletic types, gardening/caring types, and walkers. One-way ANOVA Bonferroni correction post hoc tests are significant if p <  0.05 (correction already included). Chi-square post hoc bivariate tests are significant if *p* <  0.0125 (Bonferroni correction)

### Patterns of physical activity: a four-class model

**Fig. 2:**
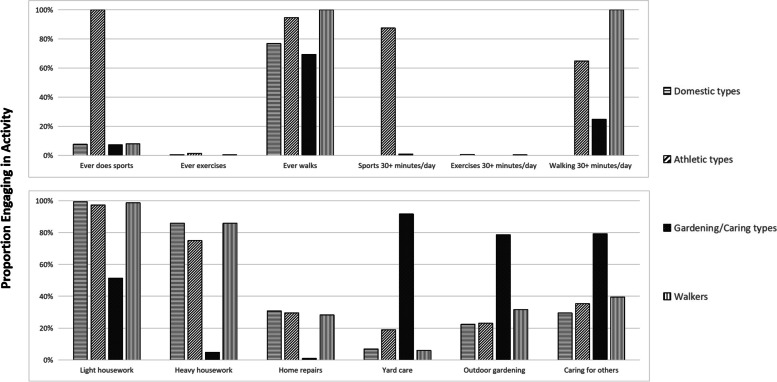
Proportion of activity reported by community-dwelling older adults in Shanghai, China, grouped by four latent classes. Note: Sports refers to sports or recreational activities such as dancing, hiking, and jogging; exercise includes muscular strength or endurance exercises (such as sit-ups and weight lifting)

Table 2 presents results from the LCA. Five latent class models were created, specifying latent class counts from two to six. According to recommendations of model selection in LCA [40, 41], we chose a four-class solution as the best fitting model. In a large sample, the sample-size adjusted BIC (aBIC) will keep decreasing with the increase of latent class counts [42]. But a four-class model showed the highest entropy, representing the highest certainty of classification. Moreover, the four-class model was interpretable and reasonably well defined (Fig. 2). The final latent classes were as follows: 1) older adults who reported housework but little other activities (29.7% of participants, “domestic types”); 2) older adults who were physically active, especially engaging in sports (11.7% of participants, “athletic types”); 3) older adults who reported yard care, gardening and caring for others but little other activities (20.9% of participants, “gardening/caring types”); and 4) older adults who reported walking and some housework (37.7% of participants, “walkers”). Fig. 2 presents the proportion of physical activities engagement of community-dwelling older adults in the condition of latent class assignment.

**Table 2:** LCA model fit statistics

Classes	AIC	BIC	aBIC	Entropy	LMR	BLRT
2	24,642.702	24,788.552	24,709.121	0.883	< 0.0001	< 0.0001
3	23,673.848	23,895.54	23,774.804	0.895	< 0.0001	< 0.0001
4	23,368.236	23,665.77	23,503.729	0.915	< 0.0001	< 0.0001
5	23,166.994	23,540.37	23,337.025	0.868	< 0.0001	< 0.0001
6	23,021.524	23,470.741	23,226.092	0.784	0.3153	< 0.0001

*Note*: AIC, Akaike information criteria; BIC, Bayesian information criteria; aBIC, sample-size adjusted BIC; LMR, *p*-value for the Lo-Mendell-Rubin likelihood ratio test; BLRT, *p*-value for the bootstrapped likelihood ratio test

### Relationship between PASE scores, patterns of PA, and depression

Patterns of PA were associated with depression: 26.9% of the gardening/caring class was depressed, compared with 13.3, 3.4, and 9.2% of the domestic types, athletic types, and walkers, respectively (χ^2^ test, *p* <  0.001). In multivariable models (Table 3), PASE scores and patterns of PA both were associated with depression, suggesting that patterns of PA provided information above and beyond PASE score alone (PASE scores, r^2^ = 0.169; patterns of PA, r^2^ = 0.178; both, r^2^ = 0.182).

**Table 3:** Logistic regression models showing the associations between PASE score, patterns of PA, and depression (n = 2525)

	Model 1	Model 2	Model 3
Variable	OR (95% CI)	OR (95% CI)	OR (95% CI)
**Standardized PASE score**	0.77** (0.66–0.91)		0.78* (0.64–0.94)
**Patterns of PA (Domestic types as reference)**			
*Athletic types*		0.27***(0.14–0.53)	0.35**(0.17–0.71)
*Gardening/Caring types*		1.25 (0.89–1.75)	1.55* (1.06–2.26)
*Walkers*		0.73 (0.53–1.01)	0.88 (0.62–1.24)

Notes: all models were adjusted for age, sex, education, income, living arrangements, self-rated health, and chronic disease. *p < 0.05; **p < 0.01; ***p < 0.001

Model 1: association between only PASE quantity and depression

Model 2: association between only patterns of PA and depression

Model 3: association between both PASE quantity, patterns of PA and depression

Controlling for sociodemographic and health-related characteristics, PASE scores were negatively associated with depression. Older adults who had higher PASE scores (standardized) were less likely to be depressed (*OR* = 0.78, 95% CI = 0.64–0.94). Patterns of PA also showed significant association with depression; compared with older adults who were domestic types, the athletic types were less likely to report depression (*OR* = 0.35, 95% CI = 0.17–0.71), while the gardening/caring types were more likely to report depression (*OR* = 1.55, 95% CI = 1.06–2.26). There were no significant differences in depression between the domestic types and the walkers.

### The joint influence of levels and patterns of PA on depression

**Fig. 3:**
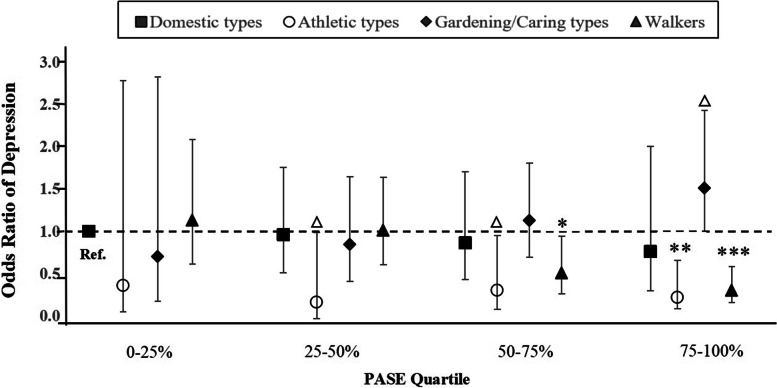
Multivariate-adjusted odds ratio of depression according to levels of PA and patterns of PA. Notes: 1) PASE: the Physical Activity Scale for the Elderly; PA: physical activity; 2) the model was adjusted for age, sex, education, income, living arrangements, self-rated health, and chronic disease; 3) PASE quartile: 0–25%, 25–50%, 50–75%, based on the participants in the study; patterns of physical activity including domestic types, athletic types, gardening/caring types, and walkers 4) the reference group was older adults with PASE quartile: 0–25% and domestic types; 5) Bars show 95% confidence intervals; *p* <  0.1; *p <  0.05; **p <  0.01; ***p <  0.001

Figure 3 presents the multivariate-adjusted odds ratio of depression according to levels of PA (PASE quartile: 0–25%, 25–50%, 50–75%, 75–100%) and patterns of PA (domestic types, athletic types, gardening/caring types, and walkers). Results were adjusted for age, gender, education, income, marital status, living arrangements, and physical health status. Older adults who were the most active (PASE quartile: 75–100%) and the athletic types had the strongest significant association with depression (*OR =* 0.19, 95% CI: 0.06–0.65), followed by those who were the most active (PASE quartile: 75–100%) and the walkers (*OR* = 0.28, 95% CI: 0.14–0.57) when compared with older adults with the least activity (PASE quartile: 0–25%) and domestic types. Older adults who were the most active (PASE quartile: 75–100%) and the gardening/caring types were most likely tend to be depressed (*OR* = 1.52, 95% CI: 0.94–2.46).

## Discussion

Our study identified four patterns of PA in community-dwelling older adults in Shanghai, China, including domestic types, athletic types, gardening/caring types, and walkers. Our study supported patterns of PA provide information above and beyond PASE score alone. Patterns of PA is a multidimensional construct consisting of types, intensity, frequency, duration and volume, which can help to understand the relationship with depression comprehensively than just by a single dimensional analysis such as PASE quantity. Significantly, patterns of physical activity appeared to be associated with depression, because independent of the total amount of PA, athletic types seemed to have the strongest association with lower risk of depression. Gardening/caring types tended to be associated with a higher risk for depression in subjects with the highest level of activity (PASE quartile: 75–100%).

To our knowledge, this is the first study that used a latent class approach to identify patterns of PA and explore their association with depression in Chinese community-dwelling older adults. Our results share several similarities with the findings of Mooney et al. and Joshi et al. [22, 35]. Mooney et al. [35] identified five patterns of physical activity in New York City residents aged 65–75: least active, walkers, athletic types, domestic/gardening athletic types, and domestic/gardening types. A different pattern, gardening/caring type was identified in our study might due to the specific cultural contexts in China. “Raising sons for old age” is a well-known Chinese saying. The young-old children take the primary responsibility of caring for their old-old parents [43]. Besides, grandparents commonly prefer to take care of grandchildren due to cultural traditions in East Asian countries [44].

Our findings supported a significant association between quantity of PA, patterns of PA and depression among community-dwelling older adults. When examining the joint effects of the quantity of physical activity and patterns of physical activity on depression risk, we found that a significant reduction in odds ratio was found only in the highest PA level, indicating that a dose-response relationship might exist. Previous research showed different effects of various dose of PA on depression, for example, a larger treatment dose of exercise might result in a great improvement in depressive symptoms [45, 46]; and light-intensity PA showed a favorable effect on depression prevention, but moderate-to-vigorous PA was not [47, 48]. Although we know little about the optimal amount of PA activity needed to reduce the risk of depression [49], our findings suggested that different patterns of PA might have different dose-response relationships with depression and that such differences were significant in the highest level of PA.

It is interesting to note that gardening/caring types tended to associate with a higher risk of depression, especially in older adults with the highest level of activities. These findings are in line with some previous studies that found caregivers were associated with higher risk of depression [50, 51]. Although caring may provide the opportunity to experience the benefits of caring [52]; however, increased hours spent caring was associated with depression, as increasing caring time may decrease time for doing more pleasurable activities [53, 54]. This may explain why an adverse effect of caring on depression was observed in the highest level of PA. Andrade-Gómez et al. [55] pointed out that walking or playing sports might be more effective in reducing the risk of depression than domestic work, such as rearing children or gardening. This further emphasized the importance of inquiring about the underlying mechanisms regarding different patterns of PA and depression.

From a physiological perspective, physical activities such as walking or playing sports may cause immune system changes by reducing some inflammatory markers [56]. It was also found that monoamines and endorphins increased after exercise [57, 58]. From psychosocial perspectives, PA may increase social interaction and enhance feelings of enjoyment, self-worth, and self-esteem [55]. Those physiological and psychosocial factors resulted in a lower risk of depression [55–58]. In line with previous studies [59–61], our findings support the benefits of exercise (walking and athletic activity) on depression. Our study suggested that domestic activities cannot replace exercise for depression prevention and that we must pay more attention to the older adults who are highly engaged in gardening and caring activities. Gardening or caring may be routine, obligatory, or repetitive [54], and with an increased level of such type of activities, pleasurable feelings may become reduced and physical exhaustion may occur [62]. It seems that both psychosocial experience and levels of PA do matter for depression. Further research is warranted to explore how different patterns and quantity of PA influence depression physiologically and psychologically.

### Strengths and limitations

One of the main strengths of this study was the use of LCA to identify patterns of PA among Chinese community-dwelling older adults, solving the problem that different types of PA may be too highly correlated to be compared directly. The results of LCA can be used to direct the development of individualized interventions for the Chinese community-dwelling older adults. Community-based service and support program can be developed targeting to older adults with specific patterns of PA. For example, to provide respite care service to older adults who are highly engaged in gardening/caring for others. Further, we used population-based data to examine the joint effects of levels and patterns of PA on depression, which expanded our understanding of the associations among doses and patterns of PA and the likelihood of depression.

Several limitations should be noted. First, this study had a cross-sectional design, which precludes inference on causality. Second, data on PA were self-reported and thus could be prone to recall bias. Levels of PA (PASE quartile) were only based on our participants. Third, although we controlled for important confounders, there could still be bias due to unmeasured confounding variables, for example, we only used the presence of disease, but did not consider some specific diseases. Fourth, our sample was from only one district of Shanghai, China; thus, further study is warranted to replicate our results.

## Conclusion

In conclusion, we found that both the quantity and patterns of physical activity are associated with depressive symptoms among community-dwelling older adults. Our findings suggested two important features to guide the development of interventions. First, population-level intervention should encourage community-dwelling older adults to increase their quantity of PA to reduce the risk of depression. Athletics and walkers are recommended. Second, to individually develop tailored interventions, more attention should be paid to older adults who are highly engaged in gardening/caring for others.

## Supplementary Information

**Additional file 1.**

## Data Availability

Our data may not be shared directly, because it is our teamwork; informed consent should be attained from all the team members. Our data or material may be available after contacting the corresponding authors.
