# Supplementary material for: Patterns of physical activity and their relationship with depression among community-dwelling older adults in Shanghai, China: a latent class approach
Source: BMC Geriatr. 2021 Oct 21;21:587. doi: 10.1186/s12877-021-02537-8 (PMC8532283; doi:10.1186/s12877-021-02537-8)
Supplement: Supplementary file 1 — Additional file 1. [file 12877_2021_2537_MOESM1_ESM.docx]

**Patterns of physical activity and their relationship with depression among community-dwelling older adults in Shanghai, China: A latent class approach**

Yan Liang ^1,†^, Xinghui Li ^2,†^, Tingting Yang ^2^, Mengying Li ^2^, Ye Ruan ^3^, Yinghua Yang ^4^, Yanyan Huang ^5,6,^, Yihua Jiang ^7,8*^, Ying Wang ^2,9,*^

^1^ School of Nursing, Fudan University, Shanghai 200032, China

^2^ Fudan University School of Public Health, Shanghai 200032, China

^3^ Shanghai Center for Disease Control and Prevention, Shanghai 200336, China

^4^ Shanghai Center for Clinical Laboratory, Shanghai 200126, China

^5^ Department of Geriatrics, Huashan Hospital Fudan University, Shanghai 200040, China

^6^ TianQiao and Chrissy Chen Institute Clinic Translational Research Center, Shanghai 200040, China

^7^ Shanghai Medicine-Mental Health Center of Minhang District, Shanghai 201112, China

^8^ Minhang Branch, School of Public Health, Fudan University, Shanghai 200032, China

^9^ Key Laboratory of Health Technology Assessment, National Health and Family Planning Commission of the People’s Republic of China, Fudan University, Shanghai 200032, China

† These authors contributed equally to this work.

* These authors are co-corresponding authors. Address correspondence to Ying Wang, PhD, Fudan University School of Public Health. 130 DongAn Road, Shanghai 200032, China.

*Correspondence to [mhjswszx@126.com](mailto:mhjswszx@126.com) (Y. Jiang); [wangying1013@fudan.edu.cn](mailto:wangying1013@fudan.edu.cn) (Y. Wang)

1. Sociodemographic and physical health status questionnaire

The questionnaire was developed for this study and used to collect sociodemographic and physical health status information.

| Age | year |
| --- | --- |
| Gender | 1 □Male 2、□Female |
| Education | 1、□Illiteracy 2、□Primary school 3、□Middle school  4、□High school 5、□College and more |
| Marital status | 1、□Married 2、□Never married 3、□Divorced  4、□Widowed 5、□Else |
| Living arrangements | 1、□Living alone 2、□Live with spouse only 3、□Live with spouse and children 4、□Live with others |
| Currently income per month, Yuan | 1、□Without income 2、□≤ 1000 3、□1001–2000  4、□2001–5000 5、□5001–10000 6、□≥10001 |
| Do you have any chronic disease? | 1、□Yes 2、□No |
| How would you describe your current health status? | 1、□Excellent 2、□Very good 3、□Good  4、□Fair 5、□Poor |
